# NF-κB-Dependent Role for Cold-Inducible RNA Binding Protein in Regulating Interleukin 1β

**DOI:** 10.1371/journal.pone.0057426

**Published:** 2013-02-21

**Authors:** Christian Brochu, Miguel A. Cabrita, Brian D. Melanson, Jeffrey D. Hamill, Rosanna Lau, M. A. Christine Pratt, Bruce C. McKay

**Affiliations:** 1 Cancer Therapeutics Program, Ottawa Hospital Research Institute, Ottawa, Canada; 2 Department of Cellular and Molecular Medicine, University of Ottawa, Ottawa, Canada; 3 Department of Biology, Carleton University, Ottawa, Canada; McGill University, Canada

## Abstract

The cold inducible RNA binding protein (CIRBP) responds to a wide array of cellular stresses, including short wavelength ultraviolet light (UVC), at the transcriptional and post-translational level. CIRBP can bind the 3'untranslated region of specific transcripts to stabilize them and facilitate their transport to ribosomes for translation. Here we used RNA interference and oligonucleotide microarrays to identify potential downstream targets of CIRBP induced in response to UVC. Twenty eight transcripts were statistically increased in response to UVC and these exhibited a typical UVC response. Only 5 of the 28 UVC-induced transcripts exhibited a CIRBP-dependent pattern of expression. Surprisingly, 3 of the 5 transcripts (IL1B, IL8 and TNFAIP6) encoded proteins important in inflammation with IL-1β apparently contributing to IL8 and TNFAIP6 expression in an autocrine fashion. UVC-induced IL1B expression could be inhibited by pharmacological inhibition of NFκB suggesting that CIRBP was affecting NF-κB signaling as opposed to IL1B mRNA stability directly. Bacterial lipopolysaccharide (LPS) was used as an activator of NF-κB to further study the potential link between CIRBP and NFκB. Transfection of siRNAs against CIRBP reduced the extent of the LPS-induced phosphorylation of IκBα, NF-κB DNA binding activity and IL-1β expression. The present work firmly establishes a novel link between CIRBP and NF-κB signaling in response to agents with diverse modes of action. These results have potential implications for disease states associated with inflammation.

## Introduction

Cold-inducible RNA binding protein (CIRBP), also known as A18 hnRNP is an 18 kDa protein of the glycine-rich RNA binding protein (GRP) family [1]. The N-terminus contains an RNA recognition motif (RRM) while the C-terminus contains a glycine-rich domain [2], [3]. The GRP family of proteins was originally identified in plants but these proteins are conserved from plant to human and they appear to retain many of their functions throughout evolutionary history [4]. Notably, many of these proteins are induced in response to hypothermia and they contribute to cold-tolerance [4]. Human CIRBP itself is highly conserved with orthologs in *Mus musculus*, *Xenopus laevis* and *Arabidopsis thaliana* sharing 98, 90 and 47% amino acid identity, respectively [1], [5]–[8]. Originally, it was proposed that cold-induced CIRBP inhibited the proliferation of murine fibroblasts, however this observation was not supported by recent experiments in CHO cells [9]. CIRBP is widely expressed in tissues that are not exposed to hypothermic conditions so the role of CIRBP in other cellular processes has recently gained attention [10], [11]

Mammalian CIRBP was also identified as an ultraviolet (UV) light inducible mRNA in Chinese hamster ovary cells [12]. Not only is CIRBP up-regulated in response to UV light and hypothermia but it responds to a wide variety of other cellular stresses including hypoxia, arsenite and 2-acetylaminofluorene treatments, suggesting that CIRBP is a more general stress responsive protein [1], [2], [11], [13]. CIRBP is regulated in part at the transcriptional level, however the subcellular localization of CIRBP protein is also subject to stress-specific regulation [14]. CIRBP is primarily nuclear in unstressed cells and remains nuclear in cells exposed to moderate hypothermia [14]. In contrast, exposure of cells to UVC results in the redistribution of CIRBP from the nucleus to the cytoplasm where it is thought to stabilize specific mRNAs and promote their translation [15]–[17]. Arsenite treatment also results in the nuclear export of CIRBP, however unlike UVC the protein is directed specifically to stress granules [11]. Collectively, CIRBP responds to a variety of stresses in different ways.

Based on the presence of RRM motifs, CIRBP was hypothesized to be an RNA binding protein [1], [2]. Yang and coworkers identified CIRBP-bound transcripts by passing mRNA isolated from UVC-irradiated colon cancer cells over immobilized CIRBP *in vitro* [15], [17]. These authors reported that CIRBP specifically bound to the 3'untranslated regions (3'UTRs) of the thioredoxin (TXN), RPA2 and Ataxia telangiectasia mutated and Rad3 related (ATR) mRNAs, increasing their half-life [15], [16]. Therefore, the UVC-induced accumulation and translocation of CIRBP from the nucleus to the cytoplasm may increase the expression of specific mRNA through increased stability of a subset of UVC-induced transcripts [15]–[17].

Here we reasoned that a CIRBP-dependent increase in mRNA stability in response to UVC would result in a measurable increase in available transcript. Therefore, we used RNA interference against CIRBP coupled with oligonucleotide microarrays to screen for novel CIRBP induced transcripts. Using this strategy, we identified IL1B as a transcript that exhibited a CIRBP-dependent pattern of expression following exposure to 254 nm UV (UVC), 290–320 nm UV (UVB), the chemotherapeutic agent cisplatin and bacterial lipopolysaccaride (LPS) but not moderate hypothermia. Conversely, forced expression of CIRBP increased IL-1β expression. Pharmacological inhibition of IκB kinase (IKK) blocked the induction of IL1B mRNA following UVC and LPS treatment suggesting that the induction of IL1B was NF-κB-dependent. Consistent with this interpretation, siRNAs directed against CIRBP reduced UVC-induced IκBα phosphorylation and NF-κB sequence-specific DNA binding activity. Collectively, these results suggest that disruption of CIRBP reduces NF-κB activity and we suggest that CIRBP may be a novel regulator of inflammation.

## Materials and Methods

### Cell culture and cell treatment

Normal human neonatal foreskin fibroblasts expressing human telomerase (NFhTrt) were obtained from Mats Ljungman (University of Michigan) [18]. These cells were maintained in DMEM supplemented with 10% fetal bovine serum (Wisent, St.-Bruno, QC) and gentamicin (5 µg/ml, Sigma-Aldrich Canada Ltd) at 37°C at 5% CO_2_.

For UV-irradiation (UVB- or UVC), medium was removed immediately prior to UV exposure. UVC treatment was performed with a germicidal bulb (Philips G30T8) at 1 J/m^2^/s while UVB exposure was performed using a F15T8.UVB bulb (UVP Inc., Uplands, CA) at 10 J/m^2^/s, as determined with a hand held UV-radiometer with UVC and UVB specific detectors (UVP Inc.). Fresh pre-warmed medium was replaced following treatment and cells were returned to the incubator for the indicated period of time.

Cisplatin (Mayne Pharma (Canada) Inc. Montreal, QC) was added to fresh pre-warmed medium to the indicated final concentration, for the indicated period of time. Bacterial lipopolysaccharide (LPS) (Sigma-Aldrich Canada Ltd.) was added to fresh pre-warmed medium to a final concentration of 0.5 µg/mL for 3 hours. LPS was removed and fresh pre-warmed medium was added for the remaining period of time. Cold shock was performed by incubating cells in a humidified tissue culture incubator at 32°C for the indicated period of time (with 5%CO_2_).

### RNA interference

Cells were counted with a ViCell XR automated cell counter (Beckman coulter) and approximately 10^6^ cells in 10 cm culture dishes were either mock-transfected or transfected with siRNA targeting CIRBP (GUACGGACAGAUCUCUGAAdTdT, Dharmacon Inc., Lafayette, CO), Il1b (On-Targetplus SMARTpool Il1B, Dharmacon Inc.) or a non-targeting control (siCONTROL^®^ Non-Targeting siRNA #1, Dharmacon Inc.) at 100 nM final concentration using Oligofectamine Reagent and Opti-MEM I (Invitrogen) according to the manufacturer's instructions. Cells were split 1 to 2, 24 hours following transfection, transfected a second time and cells were treated 48 hours later, with 20 J/m^2^ UVC, 150 J/m^2^ UVB, 10 µM of cisplatin or 0.5 µg/ml LPS and collected at the indicated times for either protein or RNA extraction. The non-targeting control siRNA did not significantly affect the expression of CIRBP or IL1B compared to mock-transfected controls (P≥0.05, single sample t test).

### Microarrays

Total RNA was collected from untreated, UVC treated or cold shocked fibroblasts that were either transfected with control or anti-CIRBP siRNA using the RNeasy Mini kit (Qiagen). Human Genome U133plus2.0 oligonucleotide arrays (Affymetrix, Santa Clara, CA) were used for expression analysis. Labeling of cDNAs and hybridation were performed at the Affymetrix Gene-Chip Facility of StemCore Laboratories at the Ottawa Hospital Research Institute (Ottawa, ON, Canada). Analysis was performed using Affymetrix Microarray Suite 6.0 software (MAS6.0). Genes were considered to be induced if they were statistically increased in treated samples (P≤0.0025,Wilcoxon signed rank test) in 2 independent experiments compared to controls by an average of two fold or more [19], [20].

### Immunoblotting

Cells were harvested, rinsed with PBS, lysed with 1% SDS, sonicated for 10 s using a microtip sonifier (VWR International Ltd., Mississauga, ON) and protein concentration determined using the BioRad Protein Assay (BioRad). Whole cell lysates were prepared for gel electrophoresis in LDS NuPage sample buffer (Invitrogen). Proteins (25 µg per lane) were separated in 4–12% Bis-Tris NuPAGE pre-cast gels (Invitrogen), transfer to PVDF membrane (Immobilon-P, Millipore) and stained with Ponceau S Red (5 mg/ml in 2% glacial acetic acid) to confirm transfer of proteins. The membrane was blocked overnight at room temperature in PBSMT-A (PBS, 5% nonfat dry milk powder, 0.05% Tween 20), incubated with primary antibody for 1 hour at room temperature diluted in PBSMT-B (PBS, 0.5% nonfat dry milk powder, 0.05% Tween20). Membranes were subsequently washed in PBST (PBS, 0.05% Tween20) and incubated with the appropriate peroxidase conjugated secondary antibody. Protein bands were visualized using SuperSignal West Pico Chemiluminescent Substrate (Pierce) and Kodak film. To visualize additional proteins with the same membrane, antibodies were stripped with Restore Western Blot Stripping Buffer (Pierce).

The primary and corresponding secondary antibodies used were a rabbit polyclonal recognizing CIRBP (cat#10209-AP, Proteintech Group, Chicago, IL) followed by Goat Anti-Rabbit IgG Peroxidase Conjugate (Calbiochem) and monoclonal AC-74 to detect β–actin (Sigma-Aldrich Canada Ltd.) followed by peroxidase conjugated goat anti-mouse IgG (Calbiochem).

### RT-PCR analysis

Total RNA was isolated by using the RNeasy Mini Kit (Qiagen) and reverse transcription was performed using First Strand cDNA Synthesis Kit (Fermentas), according to the manufacturer's instructions. RT-PCR was performed using a Lightcycler 2 (Roche Diagnostic) in the presence SYBR-green I (Molecular Probes) DNA stain. The primer pairs used to amplify the indicated cDNA were: ACTIN (GGGCATGGGTCAGAAGGAT and GTGGCCATCTCTTGCTCGA-3'); CIRBP (GGGAAGTCTGTAGATGGAC and GTAGCCACCACTCTGAC-3'); IL1B (GACAAGCTGAGGAAGATGCTGG and GTTGCTCCATATCCTGTCCCTG); IL8 (TCTTGGCAGCCTTCCTGATTTC and ACTTCTCCACAACCCTCTGCAC); TNFAIP6 (GGCAAATACAAGCTCACCTACG and TAGGCATCCCATCTTTCACTCC-3'); TXN (GGTGAAGCAGATCGAGAGCAAG and CACACTCTGAAGCAACATCCTG-3'). Oligonucleotides were synthesized by Sigma-Aldrich Canada Ltd.

### Transfection of CIRBP cDNA

The coding region of CIRBP was PCR amplified from cDNA prepared from NFhTrt cells using the following primers: ACAGATCATGGCATCAGATGAAGGCAAAC and TAAGCTTACTCGTTGTGTGTAGCGTAACTG. The PCR product was subcloned into pCDNA3-1 using the BamH1 and Not1 restriction sites to generate pCIRBP. NFhTrt cells were seeded at 50% confluence and the following day cells were transfected with 5 µg of either pCDNA3-1 or pCIRBP DNA using GeneJuice Transfection Reagent (EMD Chemicals, Gibbstown, NJ). Twenty four hours later cells were collected for ELISA.

### Enzyme-linked immunosorbent assay (ELISA)

Growth medium was removed and the adherent cells were harvested at the indicated time by scraping, washed with cold PBS and resuspended in 10 mM Tris-Cl pH 7.4. Cells were lysed by three cycles of freeze-thawing and protein concentration was determined using a Bradford Assay. One hundred µg of protein lysate were analyzed by ELISA using the Quantikine Human Il-1β/Il-1F2 Immunoassay kit (R&D Systems), according to the manufacturer's instructions. Il-1β is expressed in both pg/µg of total protein and relative to control samples.

### Electrophoretic mobility shift assays (EMSA)

One million NFhTrt cells were seeded per 10 cm dishes. The following day, cells were mock treated or transfected with either control or anti-CIRBP siRNAs. On the third day, cells were treated with 0.05 µg/mL of LPS and samples were collected 45 min later. The EMSA assays were performed as previously described [21]. Consensus NF-κB and control SP-1 oligonucleotides were purchased from Promega and used in EMSA. Where indicated, 2 µg of anti-p50 antibody (Millipore) was added 1 hour prior to EMSA to supershift p50 complexes.

## Results

### Identification of CIRBP-dependent UVC responsive transcripts

**Figure 1:**
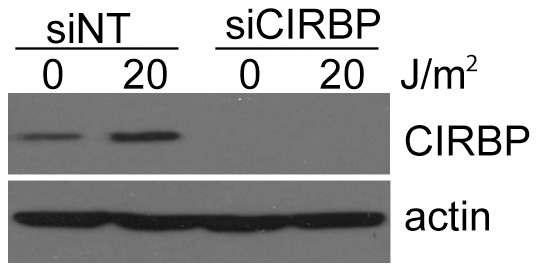
Small inhibitory RNAs targeting CIRBP inhibit the basal and UVC-induced expression of the protein. Fibroblasts were transfected with either control (NT) or CIRBP siRNAs. Seventy-two hours later, cells were exposed to 0 or 20 J/m^2^ of UVC. Whole cell lysates were collected 12 hours later for immunoblot analysis of CIRBP expression. Quantitation of this and similar immunoblots indicates that transfection of these cells with siRNA against CIRBP reduced CIRBP expression by over 80% compared to the unirradiated NT control sample (n = 5).

CIRBP was originally identified as a UVC-induced transcript in CHO cells and this was later extended to human colorectal cancer cells [2], [12], [22]. Here we confirm that CIRBP levels increase in response to UVC exposure in human fibroblasts and that transfection of these cells with siRNAs designed to target the CIRBP transcript leads to a reduction in the basal and UVC-induced levels of the protein (Figure 1). Using these siRNAs, we sought to identify CIRBP-regulated UVC-induced transcripts using oligonucleotide microarrays.

Following UVC exposure, 28 separate transcripts were significantly upregulated in the control siRNA transfected cells. As expected, the gene ontology terms ‘negative regulation of cell proliferation’, ‘apoptosis’ and ‘response to stress’ were highly represented (Table 1). Notably, 15 of these UVC-induced transcripts were reported to be p53 regulated and/or stress-responsive transcripts (Table 1) [23]. Therefore, a typical UVC response was elicited in the control siRNA-transfected fibroblasts. UV exposure of CIRBP siRNA-transfected cells resulted in increased expression of most of the same mRNAs (Table 1). Therefore, most UVC-induced transcripts were unaffected by RNAi against CIRBP.

**Table 1:** UVC-induced transcripts.

Symbol^a^	Gene ID^a^	Expression				GO Annotation^b^			p53^c^
		NT		CIRBP		Apop	Stress	-ve	
ATF3	467	5.4±1.0^d^	42.2^e^	6.6±0.4^d^	97.0^e^				○
HSPB6	126393	5.1±1.6	34.3	2.7±2.5	6.5		○		
**BTG2** f	7832	4.6±0.6	24.3	3.5±0.7	11.3	○^–^ g	○	○	○
RRAD	6234	4.3±1.1	19.7	5.1±0.9	34.3				○
GDF15	9518	3.9±0.6	14.9	4.3±1.3	19.7				○
COTL1	23406	3.9±1.3	14.9	3.4±0.1	10.6				
MAP3K4	4216	3.6±0.1	12.1	3.2±0.4	9.2		○		
**IL1B**	3553	3.4±0.9	10.6	1.0±0.2	2.0	○	○	○	
KBTBD8	84541	3.3±0.9	9.8	2.7±0.1	6.5				
TP53INP1	94241	3.3±0.2	9.8	3.0±0.4	8.0	○			○
SESN1	27244	3.2±0.6	9.2	2.3±0.1	4.9		○	○	○
GADD45A	1647	3.1±0.6	8.6	3.0±0.1	8.0	○	○		○
SAT1	6303	3.1±0.2	8.6	3.0±0.2	8.0				
**PMAIP1**	5366	3.1±0.1	8.6	3.2±0.1	9.2	○			○
TNFRSF10D	8793	3.0±0.6	8.0	2.4±0.8	5.3	○^–^			○
C10orf2	56652	3.0±0.4	8.0	2.2±0.1	4.6				
CLP1	10978	3.0±0.4	8.0	2.7±0.1	6.5				
BHLHB2	8553	2.8±1.2	7.0	2.6±0.2	6.1				
HMOX1	3162	2.8±0.9	7.0	1.8±1.1	3.5	○^–^	○	○	
HIST2H2BE	8349	2.7±0.5	6.5	2.3±0.1	4.9				
TOB1	10140	2.7±0.4	6.5	2.4±0.2	5.3			○	
**TNFAIP6**	7130	2.6±0.5	6.1	1.4±0.1	2.6		○		
PTP4A1	7803	2.5±0.5	5.7	2.2±0.6	4.6				
**IL8**	3576	2.5±0.0	5.7	1.5±0.6	2.8		○	○	
**CDKN1A**	1026	2.4±0.6	5.3	1.8±0.2	3.5	○^–^	○	○	○
**SERPINB2**	5055	2.4±0.4	5.3	1.8±0.0	3.5	○			
RSL1D1	26156	2.3±0.2	4.9	2.3±0.2	4.9				
MARCKSL1	65108	2.0±0.3	4.0	1.7±0.0	3.2				

a Symbols and Gene ID are from http://www.ncbi.nlm.nih.gov/sites/entrez?db=gene.

b GOstat analysis was performed using software hosted at http://gostat.wehi.edu.au/. Statistically overrepresented GO annotations were apoptosis (GO:0006915, P = 1.5×10^−6^), response to stress (GO:0006974, P = 1.5×10^−6^) and negative regulation of cell proliferation (GO:0008285, P = 1.3×10^−7^), and related annotations.

c Known p53-induced transcripts were identified from a recent comprehensive survey [20].

d Fold increase in expression following UV exposure is expressed as the mean (log_2_) ± standard error determined by microarray from 2 independent experiments. Samples were transfected with either control (NT) or CIRBP siRNAs.

e Fold increase in expression (see ^c^) in linear form (2^log2mean^).

f Expression of transcripts in bold were independently confirmed by q-RT-PCR.

g The symbol ‘○’ is used to designate that the indicated transcript was annotated with the indicated GO term. Within the GO term apoptosis (GO:0006915), the ‘^–^‘ superscript denotes negative regulators of apoptosis (GO: 0043066).

Nonetheless, RNA interference against CIRBP inhibited the full induction of at least 5 of these UVC-induced transcripts (IL1B, IL8, TNFAIP6, HMOX1 and HSPB6) (Table 2), consistent with its reported function [15]–[17]. In further accord with its role in stress responses, all 5 of these apparent CIRBP-regulated transcripts were associated with the gene ontology term ‘response to stress’. Unexpectedly, 3 of these 5 transcripts were also associated with the GO term 'inflammatory response' (Table 2). Our results suggest, for the first time, that CIRBP influences the expression of inflammatory molecules in response to UVC exposure.

**Table 2:** Potential CIRBP-dependent gene expression determined by microarray analysis.

Symbol a	Expression				GO Annotation b	
	NT		CIRBP		Stress	Inflammation
IL1B	3.4±0.9 c	10.6 d	1.0±0.2 c	2.0 d	○ e	○
TNFAIP6	2.6±0.5	6.1	1.4±0.1	2.6	○	○
IL8	2.5±0.0	5.7	1.5±0.6	2.8	○	○
HMOX1	2.8±0.9	7.0	1.8±1.1	3.5	○	
HSPB6	5.1±1.6	34.3	2.7±2.5	6.5	○	

a Gene symbol and ID were obtained from http://www.ncbi.nlm.nih.gov/sites/entrez?db=gene.

b GOstat analysis was performed using software hosted at http://gostat.wehi.edu.au/. Statistically overrepresented GO annotations were inflammatory response (GO:0006954, P = 1.7 10^−4^), response to stress (GO:0006950, P = 2.0×10^−5^), and related annotations.

c Fold increase in expression following UV exposure is expressed as the mean (log_2_) ± standard error determined by microarray from 2 independent experiments. Samples were transfected with either control (NT) or CIRBP siRNAs.

d Fold increase in expression (see ^c^) in linear form (2^log2mean^).

e The symbol ‘○’ is used to designate that the indicated transcript was annotated with the indicated GO term.

### CIRBP regulates IL-1β following exposure to UVC, UVB and cisplatin

**Figure 2:**
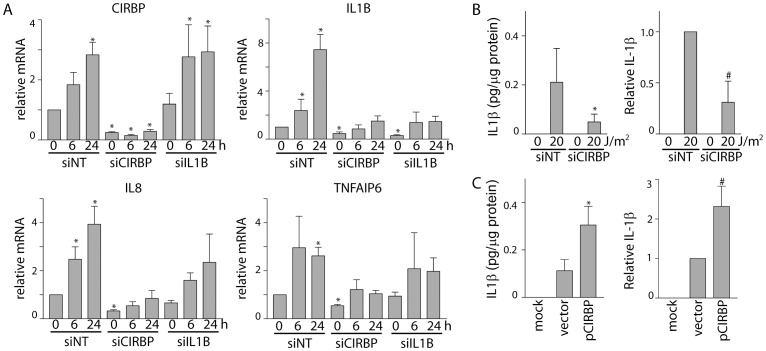
The CIRBP-dependent UVC-induced accumulation of IL1B, IL8 and TNFAIP6 mRNAs. (A) Fibroblasts were transfected with either control (NT), CIRBP or IL1B siRNAs, 72 hours later cells were exposed to either 0 or 20 J/m^2^ of UVC light and total RNA was collected at the indicated time following UVC exposure for qRT-PCR analysis of the indicated mRNAs. (B) Fibroblasts were similarly transfected with siRNAs 72 hours prior to UV exposure Cells were collected by scraping and IL-1β protein was measured by ELISA at 16 hours following UVC exposure. (C) Cells were transfected with the indicated expression vector and 24 hours later cells were collected and analyzed as described in (B). Each value in A, B and C represents the mean (± SEM) determined from a minimum of 3 independent experiments. An * indicates that the value is significantly different than similarly treated control samples (P≤0.05, t test) while a # indicates that the value is significantly different from 1.0 (P≤0.05, single sample t-test).

The possible role of CIRBP in regulating inflammatory molecules was entirely unexpected. Of note, IL-1β is a cytokine that plays a prominent role in inflammation and it can stimulate the production of IL-1β itself, IL8 and TNFAIP6 [24]–[27]. Therefore, the UVC-induced accumulation of IL8 and TNFAIP6 detected through microarray analysis may be indirect due to IL-1β expression and activity**.** To address this question, human fibroblasts were transfected with control, CIRBP or IL1B siRNAs and the effect of UV light on CIRBP, IL1B, IL8 and TNFAIP6 expression was determined using qRT-PCR. Consistent with microarray analysis, siRNAs against CIRBP reduced the UVC-induced accumulation of all of these transcripts (Figure 2A). The siRNA directed towards IL1B strongly inhibited the basal and UVC-induced levels of IL1B (Figure 2A). While CIRBP mRNA was induced in response to UVC in ILIB siRNA transfected cells, UVC treatment did not significantly induce IL8 or TNFAIP6 expression under the same conditions (Figure 2A). It is possible that IL-1β-mediated signaling contributes, at least in part, to the UVC-induced accumulation of these transcripts. For this reason, we focused our attention on IL1B.

Discordance between IL1B mRNA and IL-1β protein and/or activity has been reported [28]. This is due to significant post-transcriptional regulation and a requirement for proteolytic cleavage of pro-IL-1β to yield active protein [29]. Therefore, we sought to determine if the IL-1β protein was also induced in a CIRBP-dependent manner following UV exposure. In unstimulated cells, IL-1β protein levels were below our level of detection using an enzyme-linked immunosorbent assay (ELISA) but IL-1β protein increased dramatically and was readily detected following UV exposure in control siRNA transfected cells (Figure 2B). Although IL-1β increased somewhat in CIRBP siRNA-transfected cells following UVC exposure, the induced level was significantly lower than in controls (Figure 2B). Importantly, overexpression of CIRBP also led to an increase in the expression of IL-1β (Figure 2C). Taken together, CIRBP positively regulates and appears to be limiting for IL-1β expression.

**Figure 3:**
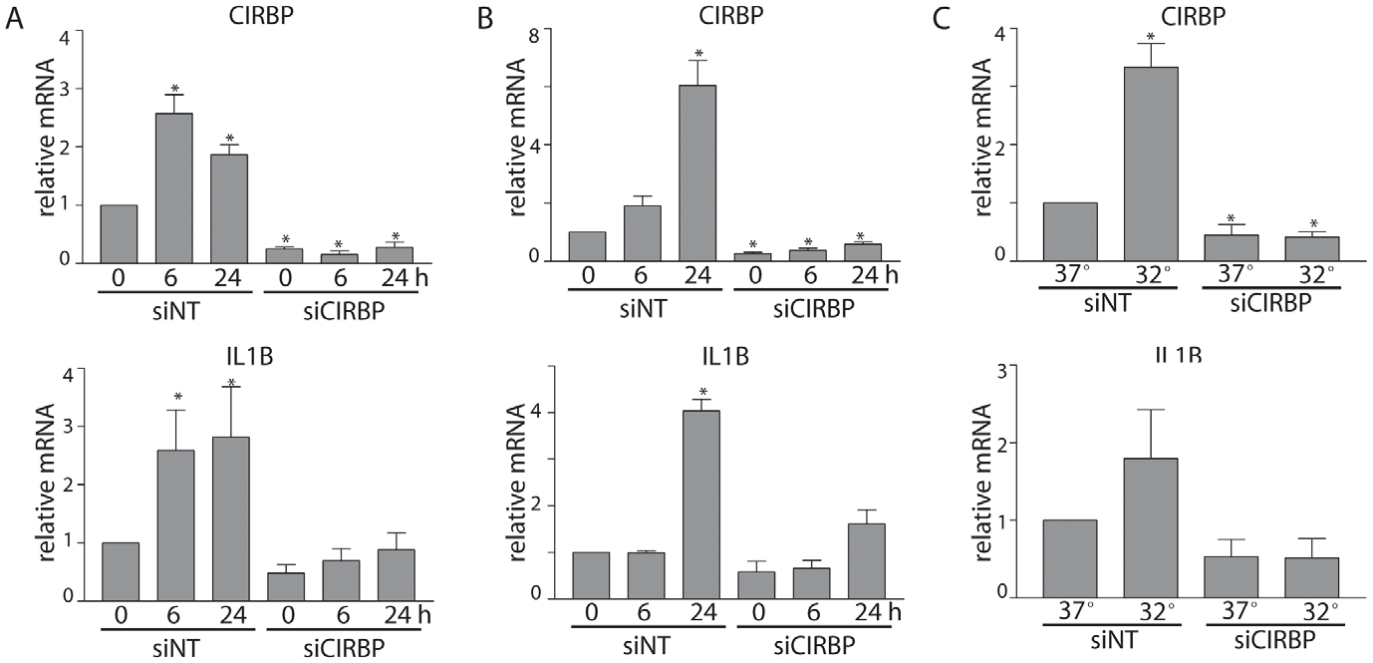
RNA interference against CIRBP prevents the induction of IL1B mRNA in response to UVB and cisplatin. Fibroblasts were transfected with non-targeting control (NT) or CIRBP siRNAs 72 hours prior to treatment with 150 J/m^2^ of UVB light (A), 20 µM cisplatin (B) or moderate hypothermia (32°C) for 24 hours (C). (A–C) At the indicated time, total RNA was collected for qRT-PCR analysis of the indicated transcript. Each value in A–C represents the mean (±SEM) determined from a minimum of 3 independent experiments. The * indicates that the mean is significantly different from 1.0 (P≤0.05, single sample t-test).

UVC is a convenient model DNA damaging agent but this wavelength of UV light is filtered by the ozone layer and does not reach the surface of the earth. Longer wavelengths of UV light (UVB) reach the earth's surface and are damaging to sun exposed skin [30]. Cisplatin is a commonly used chemotherapeutic drug that induces intrastrand DNA adducts that, like UVC- and UVB-induced DNA lesions, are processed by the nucleotide excision repair pathway [31]. Thus UVB and cisplatin induce DNA lesions are more relevant to human health. Here, the expression of CIRBP and IL1B was similarly assessed following exposure to 150 J/m^2^ UVB or 10 µM cisplatin. UVB and cisplatin led to increased expression of both CIRBP and IL1B in control siRNA transfected cells (Figure 3A and B). Once again, siRNAs against CIRBP inhibited the UVB- and cisplatin-induced accumulation of IL1B (Figure 3A and B). Interestingly, moderate hypothermia led to increased expression of CIRBP, as expected, but the relatively small change in IL1B levels was not statistically significantly under the present conditions (Figure 3C). Taken together, IL1B is induced in a CIRBP-dependent manner in response to several DNA damaging agents.

### Role for CIRBP in NFκB signaling

**Figure 4:**
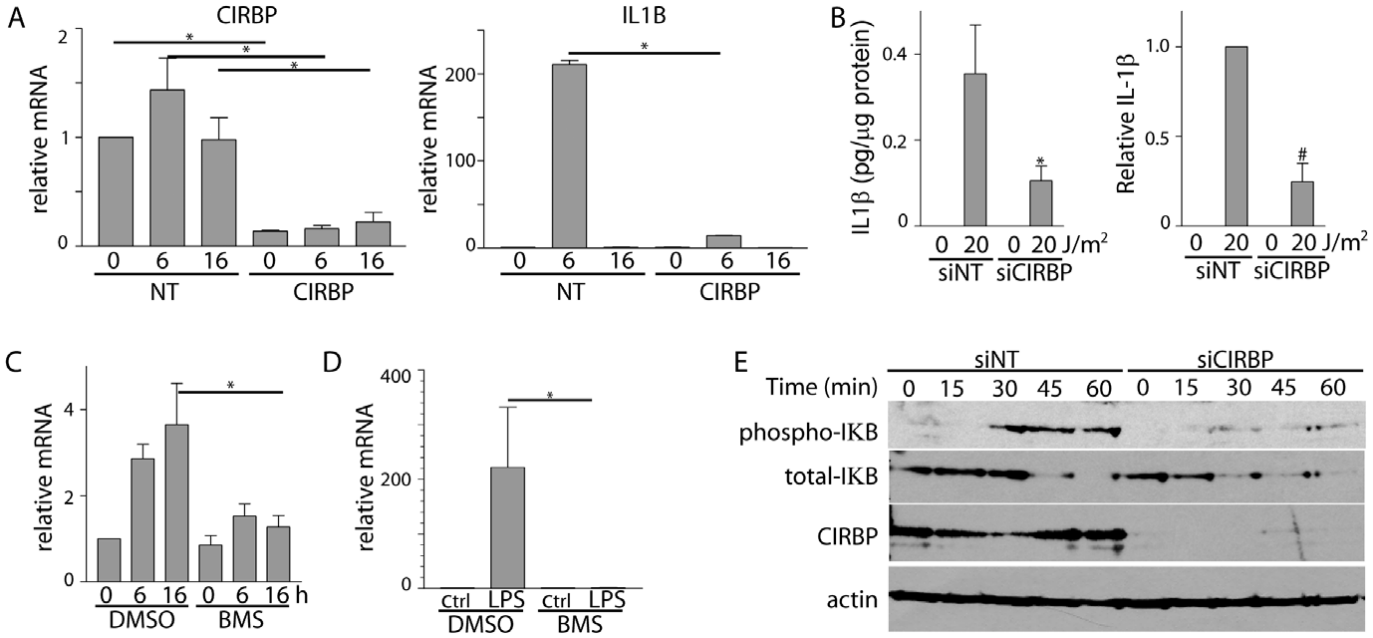
Phosphorylation of IκB is impaired in CIRBP siRNA transfected cells exposure to either UVC or LPS. (A) Fibroblasts were transfected with non-targeting control (NT) or CIRBP siRNA 72 hours prior to treatment with 0.5 µg/mL LPS. Total RNA was collected at the indicated time for qRT-PCR analysis. (B) Fibroblasts were similarly treated but IL-1β levels were assessed by ELISA 6 hours following addition of LPS. (C and D) Fibroblasts were exposed to BMS-345541 (BMS) or vehicle control at the time of cell treatment (0 or 20 J/m^2^ UVC or 0.5 µg/mL LPS). Total RNA was collected for qRT-PCR of IL1B expression at the indicated time following UVC (C) or 6 hours following LPS treatment (D). Each value in A–D represents the mean (±SEM) determined from a minimum of 3 independent experiments. An * denotes that the indicated means are significantly different from similarly treated control samples by t-test (P≤0.05) while a # indicates that the mean is not equal to 1.0 (P≤0.05, single sample t-test). (E) Fibroblasts were transfected with siRNA, as described in A, and then whole cell lysates were collected at the indicated time following LPS treatment for immunoblot analysis of phospho- IκB, total-IκB, CIRBP and β-actin, as indicated. Similar results were obtained in 4 independent experiments.

Bacterial lipopolysacharides (LPS) elicit strong immune responses through activation of the nuclear factor kappa-light-chain-enhancer of activated B cells (NF-κB). One of the key transcriptional targets of NF-κB is IL1B encoding IL-1β [32]. Here, LPS treatment did not lead to increased expression of CIRBP but LPS resulted in a remarkable increase in IL1B mRNA and IL-1β protein (Figure 4A and B). Transfection of fibroblasts with siRNAs targeting CIRBP greatly reduced the LPS-induced expression of both IL1B mRNA and IL-1β protein (Figures 4A and B). Therefore, decreased expression of CIRBP had a very pronounced affect on LPS-induced IL1B expression.

**Figure 5:**
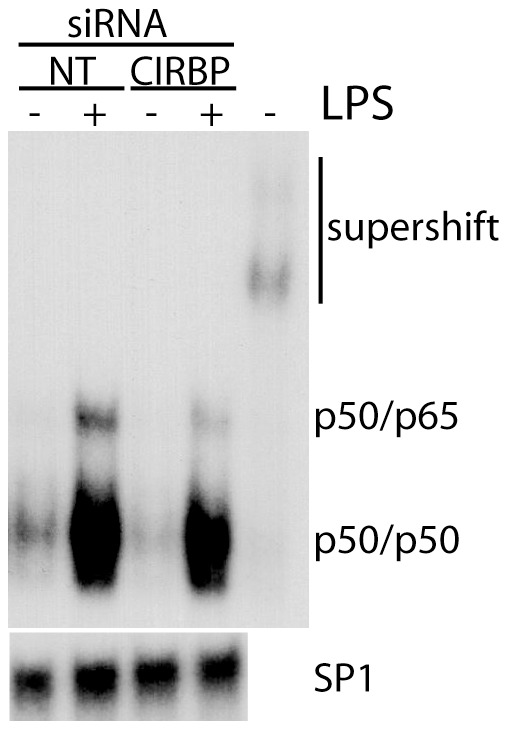
Electrophoretic mobility shift assay. Fibroblasts were transfected with the indicated siRNA 72 hours prior to a 45 min LPS treatment after which nuclear extracts were collected for electrophoretic mobility shift assays. The position of p50/p50 and p50/p65 DNA binding complexes and anti-p50 supershift complexes are indicated (upper blot). SP1 DNA binding activity was included as a loading control (lower blot). Similar results were obtained in two independent experiments.

One of the critical events in LPS-mediated NF-κB activation is the phosphorylation of inhibitor of kappa B (IκBα) by the IκB kinase (IKK-2) followed by the degradation of IκBα [33]. Therefore, the induction of IL1B was assessed in the presence of the selective IKK-2 inhibitor (BMS-345541) following UVC and LPS exposure (Figure 4C and D) [34], [35]. Consistent with a role for NFκB in IL1B upregulation, BMS-345541 significantly attenuated the induction of IL1B following exposure to either UVC or LPS. Furthermore, immunoblot analysis of LPS-treated fibroblasts indicated that IκBα was rapidly phosphorylated and degraded in response to LPS in control siRNA-transfected cells while phosphorylation of IκBα in CIRBP siRNA-transfected cells was impaired (Figure 4E). Lastly, electrophoretic mobility shift assays indicated that LPS treatment led to a specific increase in NF-κB DNA binding activity (Figure 5). Remarkably, siRNAs against CIRBP decreased the extent of LPS-induced NF-κB DNA binding activity, particularly for the p50/p65 heterodimer (Figure 5). Collectively, these results suggest that the CIRBP-dependent induction of IL1B by both UVC and LPS was dependent on IKK-2 activity, IκBα phosphorylation and thus NF-κB activity.

## Discussion

### CIRBP is involved in stress responses

Murine CIRBP was cloned from a mouse testis library in a screen to identify RNA binding proteins [1]. Comparative amino acid sequence analysis suggested that CIRBP was related to a class of stress-induced GRP in plants, several of which participate in cold-tolerance [1]. As suggested by the name, CIRBP is considered a key component of the cellular response to moderate hypothermia [1], [14] yet the role of CIRBP in the cold shock response remains enigmatic. As expected based on sequence homology, CIRBP bound RNA with a preference for polyuridine *in vitro* [1]. A cursory look at hypothermia-induced gene expression failed to identify any putative CIRBP-dependent target genes (data not shown). It is noteworthy, that unlike the UVC-response, CIRBP appears to remain localized to the nucleus in response to moderate cold stress [1], [14], so it likely serves different roles in response to these stresses. Also, very few tissues are exposed to hypothermic conditions so the ubiquitous expression of CIRBP (see files: PBB_GE_CIRBP_200810_s_at_fs.png and PBB_GE_CIRBP_200811_s_at_fs.png at http://commons.wikimedia.org/wiki/ [36]) further suggests that it is likely important for other cellular processes.

CIRBP expression was also reportedly increased in response to UVC and hypoxia [1]–[3], [12], [13], [22], [37] and here we found that CIRBP expression is increased in response to cisplatin and UVB, as well. Other investigators have reported that the localization of CIRBP can change in response to stresses [11], [15]. Notably, UVC leads to a redistribution of CIRBP from the nucleus to the cytoplasm where it is thought to stabilize specific mRNAs and stimulate their translation [15]–[17]. In partial contrast, oxidative stress, osmotic stress and sodium arsenite lead to the nuclear export of CIRBP, but under these conditions the protein is directed specifically to stress granules without a concomitant increase in CIRBP expression [11]. The following permutations have been reported: 1. increased expression without changes in localization (i.e. hypothermia), 2. increased expression with nuclear export (i.e. UVC) and 3. no change in expression but the protein is exported to stress granules (i.e. sodium arsenite). Therefore, CIRBP is a stress responsive GRP that is likely contributing to various stress responses differently through a combination of changes in protein levels and nucleocytoplasmic shuttling.

In the present work, we identified 5 transcripts that were induced in response to UVC in a CIRBP-dependent manner (IL1B, IL8, TNFAIP6, HMOX1 and HSPB6). Gene ontology analysis indicated that all 5 transcripts were associated with stress responses (Table 2). None of these transcripts had been linked to CIRBP previously but the general association with stress responses is consistent with the proposed role of CIRBP in responding to cellular stress [15]–[17], [38]. The present work further supports this general concept.

### CIRBP, inflammatory cytokines and NF-κB

Unexpectedly, three of the five CIRBP-regulated mRNAs were associated with inflammatory responses (IL-1β, IL-8 and TNFAIP6). We had anticipated that this strategy would lead to the identification of mRNAs bound directly by CIRBP [15]-[17], [38]. However, IL-1β is a key cytokine that modulates the expression of other secondary cytokines including IL-8 and TNFAIP6 [26], [27]. We found that RNAi against IL1B prevented the full induction of IL-8 and TNFAIP6 suggesting that there was an autocrine IL-1β-dependent component to their induction. Contrary to our initial hypothesis, we did not find any evidence of a direct effect of CIRBP on IL1B expression. Notably, heterologous reporter constructs containing the 3'UTR of IL1B were not stabilized in response to LPS exposure (Figure S1). Therefore, the IL1B mRNA is unlikely to be a direct target of this RNA binding protein.

The NF-κB transcription factor plays a central role in immunological responses [39]. The IL1B promoter contains NF-κB binding sites and UVC is an activator of NF-κB [40]–[42]. Here, inhibition of NFκB using a selective IKK-2 inhibitor prevented the accumulation of IL1B at both 6 and 16 hours following UVC exposure. Therefore, the increased IL1B expression here is occurring through an IKK- and NF-κB –dependent pathway, not the IKK-independent mechanisms reported previously [43], [44]. Activation of NF-κB with bacterial LPS did not increase CIRBP expression but led to massive CIRBP-dependent increases in IL1B mRNA and IL-1β protein. The increase in IL1B mRNA and IL-1β protein following LPS treatment was associated with increased IκBα phosphorylation, degradation of IκBα, increased NF-κB DNA binding activity and these changes could be blocked with BMS-345541. These changes are all consistent with activation of the canonical NF-κB response [45], indicating that CIRBP plays a role in regulating the NF-κB in response to diverse stresses without an absolute necessity for increased CIRBP expression.

NF-κB has been implicated in many cellular processes including cell survival [45]. Tumor necrosis factor alpha (TNFα) is a cytokine that can induce apoptosis through the extrinsic cell death pathway [46]. Paradoxically, TNFα is also a potent inducer of NF-κB such that TNFα is more cytotoxic when the NF-κB pathway is concurrently inhibited [45]–[47]. Interestingly, TNFα-induced apoptosis is also increased in CIRBP null MEFs while overexpression of CIRBP can stimulate NF-κB and increase survival following TNFα treatment [48]. Therefore, inhibition of CIRBP and NF-κB result in similar changes in the sensitivity of fibroblasts to TNFα. It is also noteworthy that decreased CIRBP expression is associated with increased sensitivity to UVC and that DNA damaging agents like UVC are inducers of NF-κB activity [15]. Our results support a model in which CIRBP affects the sensitivity of cells to a variety of stresses by regulating NF-κB activity.

The precise role of CIRBP in regulating NF-κB remains to be determined but recent papers provide at least one plausible link between these two proteins. CIRBP was recently reported to bind the mRNA encoding the clock circadian regulator protein (CLOCK) [49]. In turn, CLOCK is reportedly a positive regulator of NF-κB DNA binding activity and NF-κB-dependent transcription [50] so the present work is consistent with a role for CIRBP in regulating NF-κB activity and IL-1β through CLOCK expression in response to cellular stresses like DNA damage and immunological challenge.

The data presented here strengthen the concept that CIRBP is a stress responsive protein that ultimately alters the expression of other stress responsive proteins. However, the present data identify NF-κB-regulated cytokines as a class of stress responsive proteins that are abnormally regulated in the absence of CIRBP. It is noteworthy that CIRBP is highly expressed in a variety of immunological cell types (see CIRBP entry at http://en.wikipedia.org/wiki/Portal:Gene_Wiki) [36], [51]. The potential impact of CIRBP on cytokine expression was highly unexpected but the importance of this observation to inflammation *in vivo* will require testing in an animal model. CIRBP null mice have been generated but no apparent developmental phenotype was initially reported under standard laboratory conditions [48]. A detailed analysis of the CIRBP knockout mice was recently published [52]. CIRBP null mice exhibited a modest defect in spermatogenesis that appears to be associated with a role for CIRBP in the proliferation of spermatogonia [52]. Like the subtle phenotype of CIRBP-targeted mice, targeted disruption of *nfkb1* (encoding the p50 subunit of NF-κB) in mice does not lead to any apparent defects in embryonic development [53]. Instead, these mice exhibit specific defects in immune function. It will be important to determine whether additional defects are unmasked in CIRBP null mice under immunological and inflammatory stresses. The CIRBP-dependent regulation of IL-1β and NF-κB could be important in a variety of disease states including autoimmune disorders, arthritis and cancer.

## Supporting Information

Figure S1 **LPS treatment did not increased the stability of a heterologous RNA containing the 3'UTR of IL1B.**
(PDF)
