# Supplementary material for: NF-κB-Dependent Role for Cold-Inducible RNA Binding Protein in Regulating Interleukin 1β
Source: PLoS One. 2013 Feb 21;8(2):e57426. doi: 10.1371/journal.pone.0057426 (PMC3578848; doi:10.1371/journal.pone.0057426)
Supplement: Figure S1 — LPS treatment did not increased the stability of a heterologous RNA containing the 3'UTR of IL1B. (PDF) [file pone.0057426.s001.pdf]

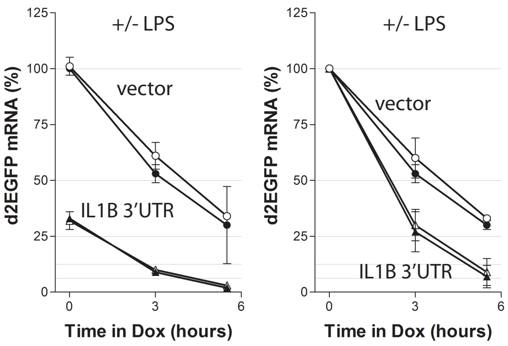

Figure S1. LPS treatment did not increased the stability of a heterologous RNA containing the 3'UTR of IL1B. The 3'UTR of IL1B was cloned downstream of the d2EGFP open reading frame that was under control of a tetracycline-regulated promoter (pTRE-d2EGFP) using a RT-PCR-based strategy and the following primers: CGGAATTCGAGAGCTGTACCCAGAGAGTC and CGGGATCCCTTCAGTGAAGTTTATTTCAG. We have used this strategy previously to study the decay of heterologous mRNAs {Melanson et al, 2011, RNA, 17, 2222}. HeLa Tet-Off® cells were transfected with either vector or the d2EGFP-IL1B and d2EGFP positive cells were identified through multiple rounds of fluorescence -activated cell sorting (FACS) using a DakoCytomation MoFlo flow cytometer (Dako, Denmark). The d2EGFP positive pools were exposed to LPS 4 hours prior to doxycycline treatment and d2EGFP RNA levels were monitored by qRT-PCR, as previously described {Melanson, 2011 #370}. The presence of the 3'UTR of IL1B led to decreased d2EGFP expression but LPS didn't increase the basal level of d2EGFP (t=0 h in left panel). Furthermore, LPS did not stabilize the heterologous reporter mRNA (right panels). Each value represents the mean +/- SEM of three determinations.
